# Shear bond strength of partial coverage restorations to dentin

**DOI:** 10.4317/jced.52471

**Published:** 2015-07-01

**Authors:** Juan-Luis Román-Rodríguez, Rubén Agustín-Panadero, Jorge Alonso-Pérez-Barquero, Antonio Fons-Font, María-Fernanda Solá-Ruíz

**Affiliations:** 1 Associate Lecturer. Department of Dental medicine, Prosthodontic and Occlusion Teaching Unit, University of Valencia, University of Valencia General Studies (UVGS), Spain; 2 Lecturer in Prosthodontics. Prosthodontic and Occlusion Teaching Unit, UVGS, Spain; 3 Assistant Lecturer. Department of Dental medicine, Prosthodontic and Occlusion Teaching Unit, UVGS, Spain; 4 Senior Lecturer. Department of Dental medicine, Prosthodontic and Occlusion Teaching Unit, UVGS, Spain

## Abstract

**Background:**

When partial coverage restorations (veneers, inlays, onlays…) must be cemented to dentin, bond strength may not reach the same predictable values as to enamel. The purpose of this study was: 1. To compare, with a shear bond test, the bond strength to dentin of a total-etch and a self-etching bonding agent. 2. To determine whether creating microretention improves the bond strength to dentin.

**Material and Methods:**

Two bonding agents were assayed, Optibond FL® (Kerr), two-bottle adhesive requiring acid etching, and Clearfil SE Bond® (Kuraray), two-bottle self-etching adhesive. The vestibular, lingual, distal and mesial surfaces of ten molars (n=10) were ground to remove all enamel and 40 ceramic samples were cemented with Variolink II® (Ivoclar Vivadent). Half the molar surfaces were treated to create round microretention (pits) to determine whether these could influence bond strength to dentin. The 40 molar surfaces were divided into four groups (n=10): Optibond FL (O); Clearfil SE (C); Optibond FL + microretention (OM); Clearfil SE + micro retention (CM). A shear bond test was performed and the bond failures provoked examined under an optical microscope.

**Results:**

O=35.27±8.02 MPa; C=36.23±11.23 MPa; OM=28.61±6.27 MPa; CM=27.01±7.57 MPa. No statistically significant differences were found between the adhesives. Optibond FL showed less statistical dispersion than Clearfil SE. The presence of microretentions reduced bond strength values regardless of the adhesive used.

**Conclusions:**

1. Clearfil SE self-etching adhesive and Optibond FL acid-etch showed adequate bond strengths and can be recommended for bonding ceramic restorations to dentin. 2. The creation of round microretention pits compromises these adhesives’ bond strength to dentin.

** Key words:**Adhesion to dentin, bonding agent, Optibond FL, Clearfil SE, microretention, shear bond test.

## Introduction

The mechanisms of bonding to enamel have been well known for some 50 years, and provide stable and predictable unions. On the contrary, bonding to dentin remains a topic for research that aims to achieve outcomes comparable to enamel (1,2).

Current dental practice is based on minimally invasive treatments often involving bonding procedures. Partial coverage restorations (veneers, inlays, onlays, overlays...) are retained by adhesion, and bond strength must be optimized and debonding avoided (3,4). Partial restorations in the anterior sector (veneers, laminates), being a more conservative treatment than full coverage restorations, are usually bonded to enamel as the main substrate. But in the posterior sector there are certain situations whereby teeth requiring inlays, onlays or overlays may have greater areas of exposed dentin than of enamel. The same can occur in the anterior sector when the teeth have suffered vestibularization or giroversion and so need greater reduction.

Generally, bonding systems for enamel based on treatment with orthophosphoric acid, a primer, and a bonding agent, or total-etch (for example Optibond FL® [Kerr, Scafaty, Italy]) are the most recommendable because of the high bond strength they achieve (2,5-7). But when the main substrate is dentin, developmental research into bond systems has pointed to self-etching adhesives as the better option.

Given the evidence that bonding onto dentin produces less strength, the use of microretentions created when the teeth are prepared could be a method for improving the adhesion of partial coverage restorations by increasing the bond surface area and through the retentive capacity of the microretentions themselves.

-Objectives

1. To compare, by means of a shear bond test, the bond to dentin of a total-etch adhesive system and a self-etching system.

2. To determine whether the creation of microretentions improves bonding to dentin.

## Material and Methods

Forty ceramic samples were fabricated (3x3 mm2) (IPS e.max Press® [Ivoclar Vivadent, Schaan, Liechtenstein]) by pressure injection.

**Figure 1:**
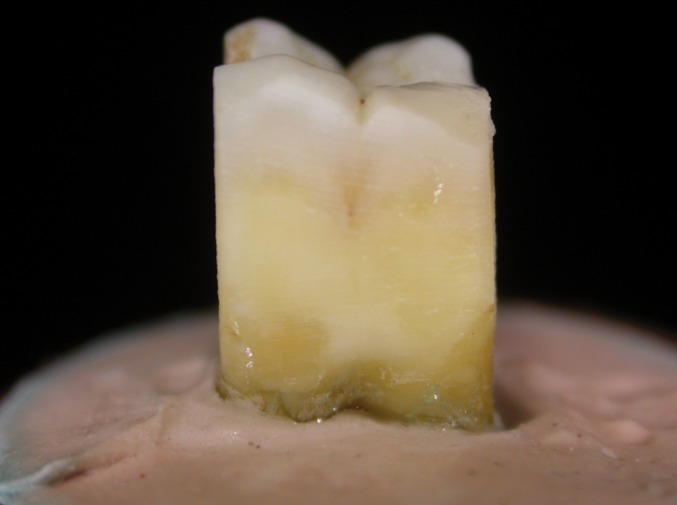
Ground dentin surface.

**Figure 2:**
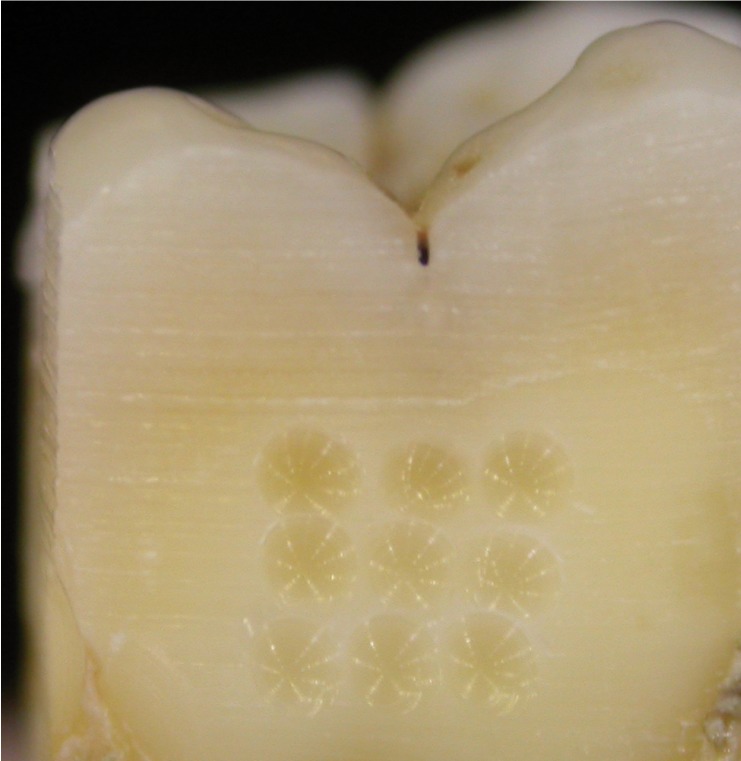
Nine pits made in the surface to be bonded (3x3 mm2).

The vestibular, lingual, distal, and mesial surfaces of ten molars (n=10) were ground down with a diamond cutting disc to eliminate all enamel (Fig. 1). For half of the surfaces (n=20), round microretentions were created (pits) in the middle of the prepared surfaces with a round diamond bur (Komet® S6801 014) (Fig. 2).

Two bonding agents were selected, Optibond FL®, two-bottle, total-etch adhesive, which has been shown to produce a reliable bond to enamel (7,8), and Clearfil SE Bond® (Kuraray, Tokyo, Japan), a two-bottle, self-etching adhesive that has shown good bond strength values to dentin (7-9) ( Table 1).

**Table 1:**
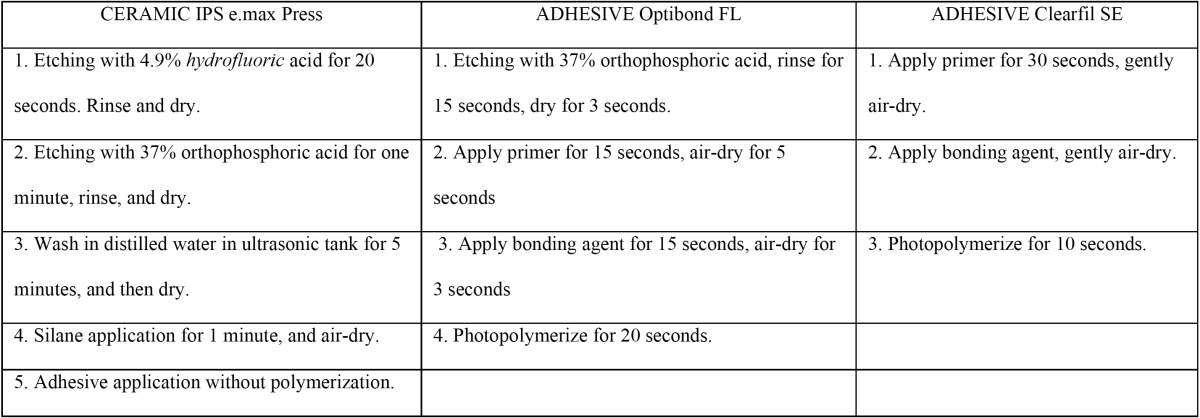
Treatment of ceramic and methods of application of each bonding agent.

Four groups of ten samples (n=10) were created: Optibond FL (O); Clearfil SE (C); Optibond FL + microretentions (OM); and Clearfil SE + microretentions (CM).

**Figure 3:**
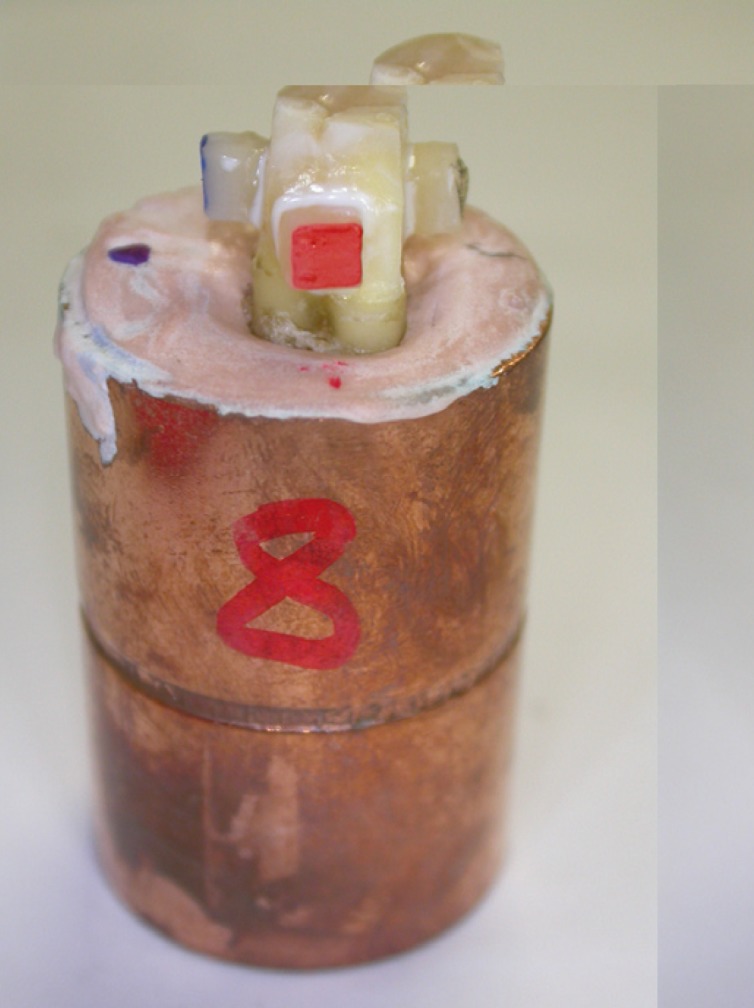
Sample molar with four ceramic blocks cemented on four sides.

All the ceramic samples were cemented with Variolink II® (Ivoclar Vivadent) resin cement with catalyst. Each molar had been prepared with four ground surfaces, and a sample from each group was cemented to each of the four sides (Fig. 3). The 10 molars were stored in a convection oven (J.P Selecta Digiheat 52L, Barcelona, Spain) for 24 hours with a humid atmosphere at 37º.

**Figure 4:**
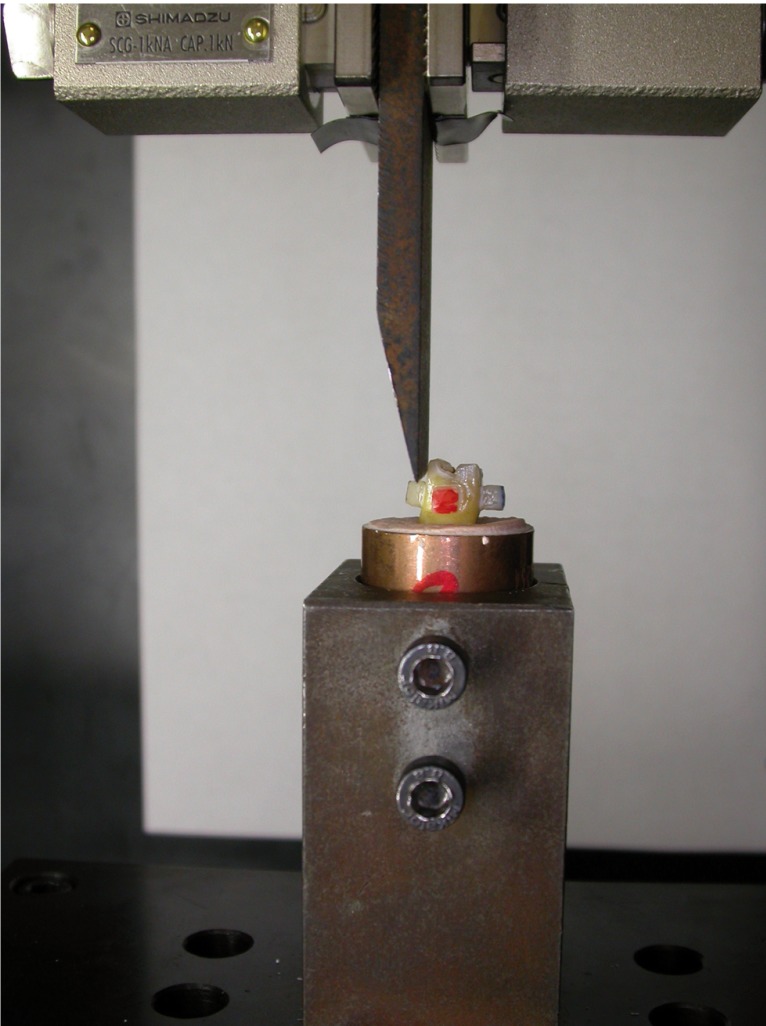
Shear bond test.

Lastly, a shear bond test was performed with a universal test machine (Shimadzu model AG-x plus®, Shimadzu corporation, Kyoto, Japan) with a cross-head speed of 0.5 mm/min. and cell charge of 1000N (Fig. 4).

-Statistical analysis

Descriptive data of shear bond strength were calculated (mean, standard deviation, range, and median) for each group. Inferential analysis consisted of estimating a Brunner-Langer non-parametric model for correlated data. An ANOVA-type model was used to evaluate the main effects and interactions. The significance level was set at 5% (α=0.05).

## Results

**Figure 5:**
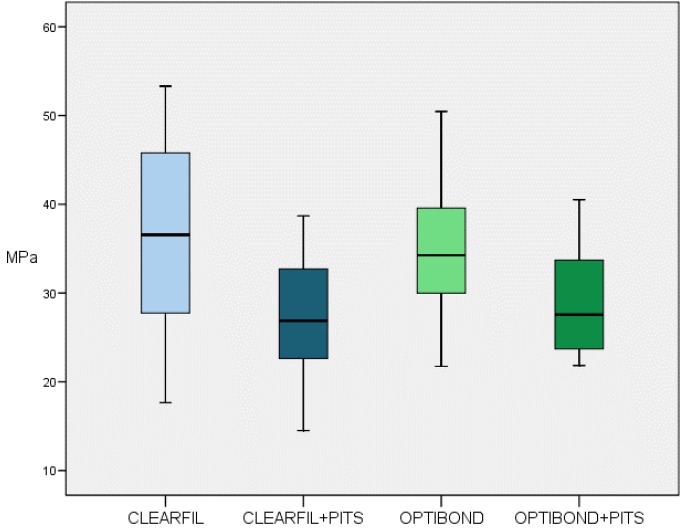
Box-plot of value distribution of bond strength (MPa) by group.

Bond strength values were similar for the two bonding agents without statistically significant differences. Optibond FL showed less statistical dispersion than Clearfil SE. The creation of microretention pits in the dentin surfaces reduced bond strength values regardless of which bonding agent was used ( Table 2,  Table 3, Fig. 5).

**Table 2:**
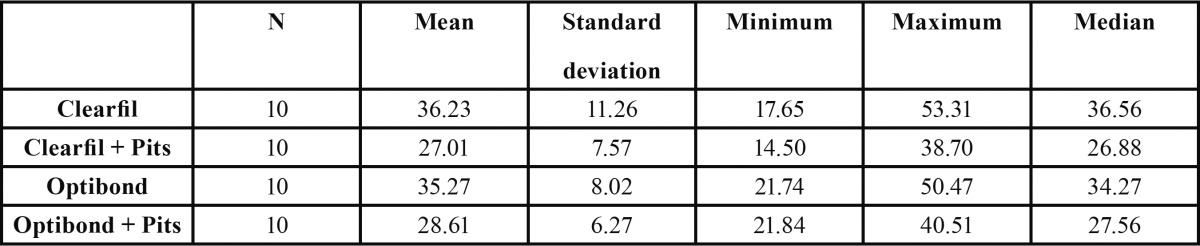
Bond strength values by group.

**Table 3:**
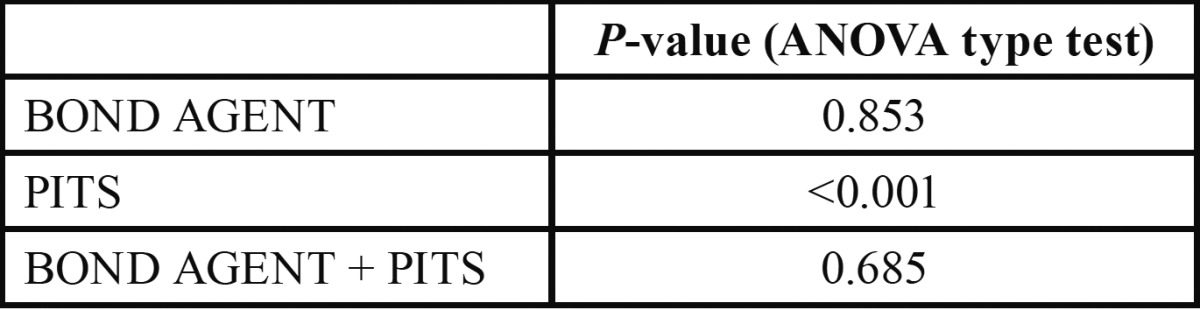
Results of ANOVA type test (ATT) for Brunner-Langer model.

## Discussion

Generally a microtensile bond strength test is preferable to a shear bond test in bond strength studies (10,11), but in the case of the present study, microtensile testing was not a viable option due to the application of microretention pits. The microtensile test method involves making cuts of very small dimensions, so that some test cuts might have coincided with a retention pit, others a section of a pit, and others would cut across an area between pits, so that the total sample would not be homogenous or representative.

The results obtained by the two bonding agents did not show statistically significant differences, although there was slightly less statistical dispersion with Optibond FL, which suggests that it offers a more predictable and homogenous performance.

Improved retention and bond strength produced by creating slots, pits, or other similar retention shapes has been investigated in a number of studies that have observed improvements of 31-81% (12,13). However, the increase in surface area and the retention capacity of the pits used in the present study did not improve bond strength at all - in fact bond strength was reduced. Two possible mechanisms might explain this reduction in bond strength. The first is the formation of pores mainly inside the pits but after examination under the optical microscope, this hypothesis was discounted. The second cause lies in the shear forces, which were very similar, and has to do with the thickness of the adhesive as the volume of adhesive penetrating into the pits could undergo fracture at lower forces and so cause the overall reduction in bond strength observed.

The bond strength values obtained coincide with those published in the literature (7,8,14). Although the work investigated two bonding systems whose bonding mechanisms are different, both were found to be equally valid as bonding agents for cases in which the main substrate is dentin (endodontically treated teeth, vestibularized teeth, cases of acute giroversion…).

-Clinical relevance 

The data obtained show that the self-etching bonding agent Clearfil SE and the acid total etch bonding agent Optibond FL produce adequate bond strength values to dentin and can be recommended for cementing ceramic restorations that need bonding to this substrate. However, the creation of microretentions in the form of round pits is not recommendable as this technique was seen to reduce the bond strength of these agents to dentin.
